# Assessment of Potato Postharvest Loss in Awi and South Gondar Zones, Amhara Region, Ethiopia

**DOI:** 10.1155/ijfo/9103840

**Published:** 2026-05-26

**Authors:** Behailu Bisenebet Mossie, Birhanu Ayitegeb Ambaw, Aynadis Molla Asemu, Wendu H/Michael H/Meskel, Mesfin Wogayehu Tenagashaw, Zenamarkos Bantie Sendekie

**Affiliations:** ^1^ Faculty of Chemical and Food Engineering, Bahir Dar Institute of Technology, Bahir Dar University, Bahir Dar, Ethiopia, bdu.edu.et; ^2^ Bahir Dar Food and Nutrition Center, Bahir Dar Institute of Technology, Bahir Dar University, Bahir Dar, Ethiopia, bdu.edu.et

**Keywords:** Amahar Region, postharvest loss, potato

## Abstract

Potatoes experience extensive physical, chemical, and physiological changes during harvesting, curing, storage, handling, shipping, and marketing, resulting in quality degradation and weight loss. The study used 540 farmer respondents, 270 for each zone, and formal questionnaires to investigate postharvest loss of potatoes in the Awi and South Gondar zones of Ethiopia′s Amhara Region. The overall postharvest loss from production (farm‐gate level) to consumption level was 205.43 and 171.51 kg/ha in the Awi and South Gondar zones, respectively. A total of 99% of farmers in the Awi Zone, as well as all South Gondar respondents, lost potatoes after harvest. The Awi Zone experienced the highest and lowest average losses of potatoes during storage (44%) and transportation (3%), while the South Gondar Zone also had the highest and lowest potato loss levels during storage (34%) and transportation (2%). Potatoes were collected manually in all research locations using conventional methods like animal plowing and hand tools, without the use of automated harvesting techniques. According to the survey, 92% of farmers in the Awi Zone and 90% in the South Gondar Zone employ traditional storage systems, while 7% and 9% use contemporary methods. In the South Gondar Zone, 100% of respondents analyzed seed storability, while in the Awi Zone, 84% did so. Gudene is the most popular cultivar for seed storage, accounting for 38% in the Awi Zone and 44% in the South Gondar Zone. However, 16% of respondents were unaware of potato varieties suited for seed storage in the Awi Zone.

## 1. Introduction

After rice and wheat, potato (*Solanum tuberosum* L.) ranks third in the world′s food crops and is the most productive vegetable crop in terms of both production and productivity [1]. More than half of the world′s harvest consists of potatoes, which are grown extensively in many developing nations [2]. In terms of production, root and tuber crops rank third among the country′s food commodities in Ethiopia, behind wheat and maize [3]. It is also one of the most extensively produced crops in the world for food and revenue [4, 5].

Preharvest loss and postharvest loss (PHL) hinder the production and sale of high‐quality produce, thereby limiting potato tuber production [6, 7]. While increasing output is one way to meet the need for food, there are fewer food crops available [8] and less money that may be made from selling these crops if PHL is not reduced [6]. The primary cause of the significant losses is the crops′ perishability, which makes them vulnerable to harm during postharvest handling procedures [9]. According to Schanes et al. [10] and Becerra‐Sanchez and Taylor [11, reducing PHL is crucial to future global food security. As a result, increasing food availability through a decrease in both quantitative and qualitative losses is necessary to maintain food and nutrition security ([12, 13, 4]; and [14]).

Potato production and consumption are crucial due to their weight and perishability [15, 16]. Harvesting, sorting, cleaning, handling, packaging, transportation, storage, distribution, and processing can all cause PHLs owing to water loss, mechanical injury, physiological deterioration, diseases, and insect damage [6, 17, 18]. PHLs impact consumers by decreasing availability and increasing commodity prices, while producers reduce their share in the price paid by consumers [19, 20].

Ethiopia has possibly the highest potential for potato production of any country in Africa [15, 21]. PHL (20%–25%) is one of the major problems in potato production [6, 22]. This indicated that the mean value of the amount of potato PHL at the producer level was 9.31 qt/year per household, which means 21.72% when we estimate it in ETB. Potato PHLs cost one household 3683.11 birr each year [20, 23]. According to Tadesse et al. [22], PHLs for producers, local traders, wholesalers, and retailers were 21.72, 1.84, 3.41, and 4.07 kg/qt, respectively.

It is therefore important that objectively evaluating the postharvest quantitative and qualitative loss be given as much attention as production practices. Reducing PHLs not only makes food available to consumers but also saves scarce resources and reduces environmental pollution caused by intensive farming [24]. Reducing PHLs, therefore, appears to be a much quicker and better way to increase the amount of food available without placing further strain on natural resources [20]. This study is aimed at estimating PHLs of potatoes in selected areas of the Amhara Region (Awi and South Gondar zones) and to identify the factors responsible for PHLs.

## 2. Material and Method

### 2.1. Descriptions of the Study Areas

This research was conducted in the Awi and South Gondar zones, Amhara Region, Ethiopia, major potato‐producing areas during the 2022/2023 and 2023/2024 growing seasons. A cross‐sectional descriptive study was conducted to assess PHLs in potatoes at the household and farm level during the harvest season. The data was collected in the Awi Zone in three woredas (Fageta, Banjia, and Guagusa) and the South Gondar Zone in three woredas (Farta, Este, and Lai‐Gaint), which are the region′s primary potato‐growing areas, accounting for 40% of potato growers [2]. The study locations were chosen with the assistance of the appropriate agricultural offices due to their high potato production, diverse farming practices, and relevance to postharvest management.

### 2.2. Sampling Technique and Sample Size

A multistage sampling approach was used to select the districts and kebeles from each zone. Districts were purposively selected based on potato production, and representative kebeles were randomly chosen from each district. Within each kebele, households participating in potato production were listed using the local agricultural office registry (sampling frame). Households were then selected using stratified random sampling, proportional to the number of potato‐producing households per kebele.

The sample size for this study was calculated based on the total population of potato producers in each zone using Cochran′s formula with finite population correction. Each zone had 900 producers, and to achieve a 95% confidence level with a 5% margin of error, a sample of 270 respondents was selected per zone:

n=n01+N0−1/N,

where *n* is the final sample size for the finite population, *n*_0_ is the sample size calculated for an infinite population, and *N* is the total population size.

The infinite population sample size, *n*_0_, is calculated based on the desired confidence level, margin of error, and estimated population proportion. The sample size of 270 respondents from each zone was considered sufficient to achieve the desired precision and confidence. This method ensures that the sample is statistically representative of the population while avoiding unnecessary oversampling.

The study interviewed 540 potential potato growers and 270 farmers in each zone to assess PHL during harvesting, transport, storage, and processing, which is determined based on Cochran′s formula for sample size estimation with a 95% confidence level, expected variability, and 10% allowance for nonresponse. Households actively engaged in potato production and storage during the study season were included, and households not cultivating potatoes or those unwilling to participate were excluded from the study. The intentional sample method was utilized, with individuals selected based on their competence. Key informants included agricultural development and cooperative offices, and 30 farmers from 18 kebeles were interviewed to learn about potato marketing and PHLs.

### 2.3. Measurement of Variables

In this study, PHL was operationally defined as the quantity of potato lost after harvesting due to factors such as handling, storage, transportation, and spoilage. The loss was estimated based on farmers′ self‐reported data and expressed in kilograms per hectare (kg/ha). Each respondent was asked to estimate the total quantity of potatoes lost from their production per unit area during the postharvest period. The reported losses were standardized to kilograms per hectare (kg/ha) to allow comparison across respondents with different farm sizes. Percentages reported in categorical tables represent the proportion of respondents who reported specific loss stages, practices, or constraints and should not be interpreted as the percentage quantity of potatoes lost.

### 2.4. Method of Data Collection

Numerators and researchers worked on the study under the direction of the primary investigator and co‐principal investigator, as well as team members. All enumerators were trained, and data were collected during the peak harvest months to capture actual PHLs. The primary data‐gathering methods were surveys, focus group discussions, key informant interviews with household heads and farm managers, and field observations of harvested potatoes, storage losses, and handling practices. Secondary data was collected from a variety of sources, including agricultural research facilities, journal papers, and the Internet. The FAO (2014) methodology was used, which comprised screening, surveying, sampling, monitoring, and problem solving [25, 26, 27].

### 2.5. Data Analysis

PHLs were calculated as a percentage of total harvested potatoes lost due to mechanical damage, pests, diseases, or storage conditions. The descriptive statistics approach of the Statistical Package for the Social Sciences (SPSS) (Version 20) software was used to evaluate the research data, including essential indicators such as percentage, frequency, mean, and comparative analysis between zones and districts. External validity was ensured through randomized household selection, while potential bias was minimized through standardization of measurement protocols and enumerator training. Categorical variables were compared using Pearson′s chi‐square (*χ* ^2^) test, and statistical significance was declared at *p* < 0.05. When expected cell counts were less than 5, Fisher′s exact test or chi‐square continuity correction was applied as appropriate. All analyses were conducted at the zonal level, and no adjustments for clustering at district or kebele levels were included in the analysis.

## 3. Result and Discussion

### 3.1. Result

The demographic characteristics of potato farmers, including age, gender, education, and household size, were assessed for 270 respondents in each of the Awi and South Gondar zones and are presented in Table 1. A total of 540 potato‐producing households were surveyed, with 270 respondents from each zone. The mean age of household heads was 50.86 ± 9.71 years in Awi and 49.12 ± 9.57 years in South Gondar, and this difference was not statistically significant (*t* = 1.68, *p* = 0.09). The average family size was 5.87 ± 1.60 in Awi and 6.25 ± 1.60 in South Gondar, and the difference was statistically significant (*t* = 2.42, *p* = 0.016). Regarding categorical variables, 57% of respondents in Awi and 54% in South Gondar had no formal education, and the majority of respondents were married in both zones (96.3% and 94.1%, respectively). Chi‐square tests indicated no statistically significant differences between the two zones in gender (*χ* ^2^ = 5.32, *p* = 0.07), educational status (*χ* ^2^ = 2.89, *p* = 0.41), or marital status (*χ* ^2^ = 2.10, *p* = 0.55). These findings indicate that the two study zones were generally comparable in most sociodemographic characteristics, with the exception of household family size, which showed a statistically significant difference.

**Table 1:** Demographic characteristics of farmers in the Awi and South Gondar zones (*n* = 270 per zone).

Description	Category	Awi, *N* (%)	95% CI	South Gondar, *N* (%)	95% CI
Gender	Men	263 (97)	94–99	251 (93)	89–96
	Female	7 (3)	1–6	19 (7)	4–11
Education status	No education	155 (57)	51–63	146 (54)	48–60
	Primary education	96 (36)	30–41	103 (38)	32–44
	Secondary education	18 (7)	4–11	19 (7)	4–11
	Above secondary education	1 (0)	0–2	2 (1)	0–3
Marital status	Single	0 (0)	0–1	0 (0)	0–1
	Married	260 (96)	92–98	254 (94)	90–97
	Widow	10 (4)	2–7	13 (5)	3–8
	Divorced	0 (0)	0–1	3 (1)	0–3
Age (years)	—	50.86 ± 9.71	49.7–52.0	49.12 ± 9.57	48.0–50.3
Family members	—	5.87 ± 1.60	5.68–6.06	6.25 ± 1.60	6.06–6.44

The market outlets used by potato farmers and their satisfaction with prices were assessed for 270 respondents in each of the Awi and South Gondar zones, providing insights into farmers′ marketing practices and perceived income adequacy Table 2.

**Table 2:** Market outlet and price satisfaction of potato farmers in the Awi and South Gondar zones (*n* = 270 per zone).

Response/place	Awi, *N* (%)	95% CI	South Gondar, *N* (%)	95% CI
Farm gate market	11 (4)	2–7	6 (2)	1–4
Village market	2 (1)	0–3	2 (1)	0–3
Town market	232 (86)	81–90	184 (68)	62–73
Farm and town	7 (3)	1–6	25 (9)	6–13
Farm and village	1 (0)	0–2	7 (3)	1–6
Farm, village, and town	1 (0)	0–2	1 (0)	0–2
Village and town market	16 (6)	4–10	45 (17)	13–22
Satisfied with market price	235 (87)	82–91	231 (86)	81–90
Not satisfied with market price	35 (13)	9–18	39 (14)	10–19
Total	270 (100)	—	270 (100)	—

Data on harvesting methods, postharvest challenges, and the prevalence of mechanical damage among potatoes were collected from 270 farmers in each of the Awi and South Gondar zones to assess practices and factors contributing to PHLs; results are presented in Table 3.

**Table 3:** Potato harvesting methods and reported problems during harvest in the Awi and South Gondar zones (*n* = 270 per zone).

Description	Response/method	Awi, *N* (%)	95% CI	South Gondar, *N* (%)	95% CI
Methods used for potato harvest	By hand tools	81 (30)	25–35	67 (25)	20–30
	By animal plowing	72 (27)	22–33	32 (12)	8–17
	By hand tools & animal plowing	117 (43)	37–49	171 (63)	57–69
Problems during harvest (multiple response)	Mechanical injury	85 (32)	26–38	55 (20)	15–25
	Cutting/scrapping	2 (1)	0–3	18 (7)	4–10
	Spoilage at farm level	28 (10)	7–14	26 (10)	6–14
	Any harvest‐related problem reported	155 (57)	51–63	171 (63)	57–69
Mechanical damage observed	Yes	255 (94)	90–97	270 (100)	98–100
	No	15 (6)	3–10	0 (0)	0–1
Total respondents	—	270 (100)	—	270 (100)	—

*Note:* “Problems during harvest” is a multiple‐response question; therefore, percentages represent the proportion of respondents reporting each problem, and respondents may report more than one category.

Distribution of PHL stages, contributing practices, and immediate farm‐level loss experience among potato producers in Awi and South Gondar, with the findings, is summarized in Table 4. This presents losses occurring immediately after harvesting at the farm level, reflecting farmers′ direct experiences at the point of harvest. The values represent respondents reporting losses specifically during harvesting, without considering subsequent postharvest stages.

**Table 4:** Stages of postharvest loss, contributing practices, and loss occurrence immediately after harvesting at the farm level in Awi and South Gondar (*n* = 270 per zone).

Variable	Category/response	Awi, *N* (%)	95% CI	South Gondar, *N* (%)	95% CI
Stages of postharvest loss	No postharvest loss	4 (2)	1–5	0 (0)	0–1
	During harvesting	112 (42)	36–48	85 (32)	27–38
	During transportation	6 (2)	1–5	4 (2)	1–4
	During storage	37 (14)	10–19	66 (24)	19–29
	During marketing	1 (0)	0–2	0 (0)	0–1
	During harvesting & storage	104 (39)	33–45	101 (37)	31–43
	During harvesting & transportation	6 (2)	1–5	13 (5)	3–8
Reasons for no experience of PHL	Lack of storage	1 (0)	0–2	0 (0)	0–1
	Family immediate use	3 (1)	0–3	0 (0)	0–1
	Experienced PHL	266 (99)	96–100	270 (100)	98–100
Practices contributing most to PHL	During harvesting	55 (20)	15–25	57 (21)	16–26
	During transportation	7 (3)	1–6	6 (2)	1–4
	During storage	120 (44)	38–50	93 (34)	28–40
	During harvesting & transportation	31 (12)	8–16	33 (12)	8–17
	During harvesting & storage	57 (21)	16–26	81 (30)	25–36
Experience of loss immediately after harvesting (farm level)	Yes	266 (99)	96–100	270 (100)	98–100
	No	4 (2)	1–5	0 (0)	0–1
Total	—	270 (100)	—	270 (100)	—

Table 5 summarizes cumulative PHLs occurring over time during storage, handling, transportation, and marketing stages. The values, therefore, reflect aggregated loss experiences across multiple postharvest stages rather than losses at a single point in time.

**Table 5:** Potato storage practices and storage‐related problems in Awi and South Gondar zones (*n* = 270 per zone).

Variable category	Response	Awi, *N* (%)	95% CI	South Gondar, *N* (%)	95% CI
Experience of postharvest loss by storage	Yes	268 (99)	96–100	270 (100)	98–100
	No	2 (1)	0–4	0 (0)	0–1
Storage practices	No storage	2 (1)	0–4	1 (0)	0–2
	In the soil	105 (39)	33–45	82 (30)	25–36
	In a bright room	98 (36)	30–42	64 (24)	19–29
	In a dark room	37 (14)	10–19	80 (30)	25–36
	DLS storage	20 (7)	4–11	25 (9)	6–13
	Basket storage	3 (1)	0–4	17 (6)	4–10
	Cold storage	4 (2)	1–5	0 (0)	0–1
	In soil & bright room	1 (0)	0–2	1 (0)	0–2
Storage problems	Deterioration	54 (20)	15–25	29 (11)	7–15
	Spoilage	71 (26)	21–32	47 (17)	13–22
	Insect attack	87 (32)	26–38	98 (36)	30–42
	Moisture loss	6 (2)	1–5	6 (2)	1–5
	Spoilage & insect attack	24 (9)	6–13	45 (17)	13–22
	Deterioration & spoilage	28 (10)	6–14	45 (17)	13–22

Information on seed storability, potato varieties cultivated, and associated PHLs was collected from 270 farmers in each of the Awi and South Gondar zones, and the results are presented in Table 6.

**Table 6:** Seed storability, potato varieties, and postharvest loss in the Awi and South Gondar zones (*n* = 270 per zone).

Variable category	Response	Awi, *N* (%)	95% CI	South Gondar, *N* (%)	95% CI
Analyzed varieties for seed storability	Yes	226 (84)	79–88	225 (83)	78–87
	No	44 (16)	12–21	45 (17)	13–22
	Total	270 (100)	—	270 (100)	—
Varieties with longer storability	Jalene	41 (15)	11–20	42 (16)	12–21
	Gudene	102 (38)	32–44	119 (44)	38–50
	Tolcha	30 (11)	7–16	26 (10)	6–14
	Local	53 (20)	15–25	40 (15)	11–20
	I do not know	44 (16)	12–21	43 (16)	12–21
	Total	270 (100)	—	270 (100)	—
Total postharvest loss (kg/ha)	—	205.43 ± 154.48	190–221	171.51 ± 127.60	159–184
Mechanical damage loss (kg/ha)	—	82.28 ± 78.52	73–91	69.61 ± 63.36	63–76

## 4. Discussion

### 4.1. Demographic Characteristics

The sociodemographic characteristics of the surveyed households indicate that the two study zones were generally comparable in most attributes, which strengthens the validity of subsequent comparisons. Although the mean age of household heads did not differ significantly between Awi and South Gondar, the observed difference in average family size was statistically significant, with South Gondar reporting slightly larger households. This may suggest potential differences in household labor availability or demographic structure between the two zones. The high proportion of respondents without formal education in both areas reflects the predominantly rural and subsistence‐based nature of potato production in the study regions and is consistent with findings from similar agricultural contexts.

The absence of significant differences in gender, educational status, and marital status suggests relatively similar socioeconomic characteristics across the study sites, which may help reduce potential confounding effects in comparative analyses. Overall, these findings suggest that observed differences in subsequent production and postharvest variables are unlikely to be strongly driven by major sociodemographic disparities, although differences in household size may have some influence on labor‐related activities.

### 4.2. Market Place of Potato

The marketplaces where farmers sell their potatoes are presented in Table 2. The producers sell their produce at varying frequencies and in a variety of market locations. The results indicate that the majority of farmers in both Awi (86%) and South Gondar (68%) zones sold their potato produce primarily at town markets, suggesting that town markets serve as the dominant marketing channels in the study areas. A relatively smaller proportion of farmers used multiple market outlets, such as village and town markets (6% in Awi and 17% in South Gondar), indicating some level of market diversification, particularly in South Gondar. The results indicate that there is a statistically significant association between market outlet and zone (*χ* ^2^ = 64.72, *p* < 0.001), suggesting that farmers in Awi and South Gondar differ in their reported choice of market outlets. Specifically, a higher proportion of farmers in Awi sold their produce at town markets, while South Gondar farmers showed relatively greater diversification across multiple market outlets.

Farmers sold their crops at varying prices depending on the location and season. Among the 270 farmer respondents from each research location, 235 (87%) from the Awi Zone and 231 (86%) from the South Gondar Zone reported being satisfied with the price of potato tubers, whereas 13% and 14% were dissatisfied, respectively, which may reflect market inefficiencies, price fluctuations, or limited bargaining power [27]. These findings are consistent with Ethiopian value‐chain studies suggesting that market proximity and infrastructure influence price satisfaction and revenue [28]. Improving market infrastructure and aggregation points may help improve price realization and reduce PHLs due to delayed sales. Overall, the findings suggest that while most farmers rely on town markets and are generally satisfied with prices, there is still room for improving market access and price competitiveness. There was no statistically significant difference in price satisfaction between the two zones (*χ* ^2^ = 0.32, *p* = 0.57), indicating that farmers in both Awi and South Gondar experienced similar levels of satisfaction with potato selling prices. This suggests that despite differences in market channels, perceived price outcomes are relatively consistent across the zones.

### 4.3. Harvesting Mechanisms of Potato Tuber

Potatoes in all the study areas were harvested manually using country plows or spades. Methods used for potato harvest and problems that were faced during harvesting regarding loss of potatoes are presented in Table 3. Farmers in the Awi Zone harvested their potato tubers by hand tools (spades) (30%), animal plowing (27%), and both hand tools (spades) and animal plowing (43%). The results indicate that the dominant harvesting method in both zones was the combined use of hand tools and animal plowing, accounting for 43% in Awi and 63% in South Gondar, suggesting a reliance on traditional and semimechanized practices. The rest of the farmers in South Gondar used hand tools (25%) and animal plowing (12%) for potato tuber harvesting methods. Those harvesting mechanisms imply that there is no mechanical harvesting method adopted for harvesting potato tubers in both study areas. The result revealed that harvesting methods significantly differed between the two zones (*χ* ^2^ = 25.84, *p* < 0.001), indicating variation in the adoption of harvesting practices. South Gondar farmers relied more heavily on the combined use of hand tools and animal plowing, whereas Awi farmers showed relatively more use of individual methods.

Regarding postharvest problems, mechanical injury was the most commonly reported issue in both zones, although a higher proportion of farmers in Awi (32%) experienced this problem compared to South Gondar (20%). The causes of potato losses during harvesting included spoilage at the farm level (10%) and mechanical damage due to scraping (1%) and mechanical damage due to scraping 20%, 7%, and 10% respectively. Respondents reported that mechanical damage to potatoes during harvesting was the most frequent and prevalent problem regarding potato loss, which accounts for 94% and 100% in the Awi Zone and the South Gondar Zone, respectively, consistent with prior Ethiopian studies showing manual handling as a major cause of tuber bruising [22]. Training farmers on gentle harvesting techniques and the provision of simple handling tools could reduce mechanical losses.

This indicates that, as observed by the respondents, potatoes are lost by mechanical damage during harvesting in both study zones. This suggests that mechanical damage is an important factor contributing to PHLs in both zones and highlights the need for improved harvesting techniques and tools to help minimize losses. There was a significant association between postharvest problems and zone (*χ* ^2^ = 48.67, *p* < 0.001), suggesting that the type and frequency of losses experienced during harvesting varied across locations. In particular, South Gondar exhibited a higher proportion of multiple combined problems.

### 4.4. PHL of Potato

Stages for PHL and practices that contribute much to the PHL of potato tubers are presented in Table 4. The findings indicated that PHLs of potatoes were mainly reported during harvesting and combined harvesting and storage stages in both zones. In Awi, 42% of respondents reported losses occurring during harvesting, while 38.5% reported losses during both harvesting and storage. In South Gondar, the corresponding figures were 32% and 37%, respectively. Storage‐related losses were also more frequently reported in South Gondar (24%) compared to Awi (14%), suggesting relatively greater storage‐related challenges in that zone.

Among the total respondents, a very high proportion reported experiencing PHLs at one or more stages. However, the magnitude of reported losses varied by stage, with higher values observed during storage and lower values during transportation in both zones. In Awi, storage‐related losses accounted for 44%, while transportation accounted for 3%. Similarly, in South Gondar, storage and transportation accounted for 34% and 2%, respectively.

Nearly all respondents in both Awi (98.5%) and South Gondar (100%) reported experiencing PHLs after harvesting (*χ* ^2^ = 4.03, *p* = 0.045), indicating that PHL is widely reported in the study areas. A small proportion of respondents in Awi (about 1%) reported no PHL, mainly due to immediate household consumption or lack of storage facilities. Among 270 farmer responders from the Awi Zone, 1% had no experience with potato loss after harvesting due to a lack of storage facilities and a family′s immediate need for eating (1%). The analysis revealed that the stage at which PHL occurs significantly differs between the Awi and South Gondar zones (*χ* ^2^ = 18.96, *p* = 0.004), suggesting variation in the distribution of losses across harvesting, storage, and combined stages. Specifically, respondents in Awi more frequently reported losses during harvesting and handling, whereas those in South Gondar more commonly reported storage‐related and combined‐stage losses.

### 4.5. Storage of Potatoes and Related Problems

Harvested potatoes are cured in the shade to adjust to the environment and heal injuries normally caused during harvesting, handling, and transportation from the field to the farmer′s home [29]. Different methods of curing were practiced in different study areas [30]. Harvested potatoes are carried directly to homes, spread in sheds, and kept for a week for curing [31]. The observed curing method appears consistent with recommended practices, which may help reduce PHLs in Awi and South Gondar. The potato storage mechanisms and the problems in both study areas are presented in Table 5. About 99.3% of farmer responders in the Awi Zone lost potato after harvesting and stored it when market circumstances were unfavorable, whereas 1% did not keep their potato seed and did not lose potato after harvesting. All farmer respondents in South Gondar experienced loss of potato after harvesting and stored their potato when market conditions were poor.

About 92% of farmers in the Awi Zone and 90% in the South Gondar Zone stored potatoes at home by using traditional storage methods, respectively, and the rest of the farmers consumed and sold their whole quantity of potatoes in the harvesting season. The rest of the farmers, 7% in the Awi Zone and 9.% in the South Gondar Zone, used modern storage by using the modified diffused light storage (DLS) method. Degebasa [6] reported that PHL of traditional storage of potatoes was as high as 29% for only 2 months in the absence of storage technologies for seed, and farmers kept potato harvest in the field for an extended period in Ethiopia. In the two zones, farmers stored the potato tuber by traditional methods at the house level by using soil, bright rooms, dark rooms, and basket storage. Regarding the storage mechanisms from the respondent farmers, 39% and 30% stored their potatoes in the soil, 36% and 24% in the bright room, 14% and 30% in the dark room, and 1% and 6% in a basket in the Awi and South Gondar zones, respectively. Storage losses ranged from 14% to 30%, similar to other African reports where lack of proper storage leads to spoilage and insect infestation [32]. Adoption of modified storage technologies (e.g., DLS and ventilated dark rooms) could reduce spoilage and extend shelf life.

The investigation reveals that traditional storage practices expose the tubers to insect attack, which significantly reduces tuber yield and quality [33]. In the traditional method, farmers store potatoes at home by stacking them on the earthen floor of dwelling houses or on bamboo‐ or wood‐made platforms for better aeration. Farmers did not store potatoes in a separate storehouse to avoid extra storage costs [34]. Generally, bulk potatoes are stored traditionally for 3–4 months [35]. In this storage method, farmers cannot maintain appropriate temperature and humidity, which in turn causes large‐scale damage to potatoes due to rot by disease, insect damage, spoilage, deterioration, and weight loss [36]. Among the 540 farmer respondents, 32% and 36% in Awi and South Gondar, respectively, said that the greatest damage to potatoes was caused by insects attacking potato tubers. The remaining damages were 26% and 17% by spoilage, 20% and 11% by deterioration, and the rest by a combination of those problems during storage in Awi and South Gondar, respectively. Generally, the findings suggest that traditional storage practices, coupled with insect infestation and spoilage, are the major contributors to storage losses in both zones. The situation appears relatively more considerable in South Gondar due to the higher prevalence of combined storage problems and lack of improved storage technologies. These results highlight the need for improved storage infrastructure, better pest management, and farmer training to reduce PHLs and enhance potato quality. The analysis shows that there is a statistically significant association between storage place and zone (*χ* ^2^ = 33.58, *p* < 0.001), indicating that farmers in Awi and South Gondar differed significantly in their choice of storage methods and storage‐related problems (*χ* ^2^ = 29.74, *p* < 0.001). Insect attack was the dominant problem in both zones.

### 4.6. Potato Variety and Storability

Storability, defined as the ability of potato tubers to remain practically edible and marketable over a relatively long period of time with minimum losses [37], is a function of tuber dormancy, respiration rate, evaporation, and infection by micro‐organisms [29]. Maintaining seed in good physical or physiological condition from the time they are harvested meaningfully enhances storability [37]. These factors are greatly influenced by temperature, relative humidity, and genotype [37]. The majority of farmers in both Awi (84%) and South Gondar (83%) (*χ* ^2^ = 0.013, *p* > 0.908) reported that they had analyzed potato varieties for seed storability, while 16% of respondents from the Awi Zone and 17% from the South Gondar Zone did not have any information about potato varieties for seed storability. Previous studies support that varietal selection significantly affects storage losses, with improved varieties outperforming local varieties in both shelf life and resistance to storage pests (Abebe Chindi [6]). This indicates that most farmers are aware of the importance of selecting varieties with better storage potential, which is crucial for reducing PHLs and ensuring seed quality for the next season. There are different varieties of potatoes available for farmers for cultivation, but the degree of selection based on storage stability varies [40].

Among the varieties reported to have longer storage life, Gudene was the most frequently cited in both zones (38% in Awi and 44% in South Gondar). It is the adoptable variety of potato tubers used by most farmers. Local varieties were also recognized, particularly in Awi (20%) compared to South Gondar (15%); there was no significant difference between zones (*χ* ^2^ = 1.632, *p* > 0.20). Other improved varieties, such as Jalene and Tolcha, were less commonly reported, suggesting either limited cultivation or lower awareness among farmers. A substantial portion of respondents (16% in Awi and 16% in South Gondar) did not know the best variety of potato for long storability, highlighting a knowledge gap regarding the storage potential of different varieties. In the Awi and South Gondar zones, there were, on average, 205.43 and 171.51 kg of potato losses per hectare, respectively, by different mechanisms. The mean PHL was statistically significantly different (*p* > 0.05), higher in Awi than in South Gondar. These values are comparable to other studies in Ethiopia, where potato PHL has been reported between 15% and 30% of the harvest [22]. The large standard deviations indicate substantial heterogeneity in farmer practices, which may be due to differences in storage practices, variety choice, or local environmental conditions. Loss due to mechanical damage also followed the same trend, with Awi experiencing higher average losses (82.28 ± 78.52 kg/ha) compared to South Gondar (69.61 ± 63.36 kg/ha). Across Africa, studies indicate that mechanical damage during harvesting and inadequate storage conditions are the primary contributors to PHLs in potato production [41]. These losses reduce both the quantity and quality of marketable tubers, directly affecting farmers′ income and food availability. This suggests that mechanical handling during harvesting, transport, or storage contributes significantly to overall PHLs and may be an area for targeted interventions such as training or improved storage/handling equipment. Mechanical damage appears to be the major contributor to losses, consistent with findings by [42]. This may be due to manual handling practices in the region.

## 5. Conclusion

PHL is a global issue, particularly in Ethiopia, where potato production has tremendous potential. To reduce it, producers should use acceptable types and current storage methods, develop processes, build market chains, promote awareness, and create loss control standards. We measured potato PHL in Ethiopia′s Amhara Region′s Awi and South Gondar zones, finding significant problems with potato production and transportation systems. These losses not only diminish the number of potatoes available for consumption and sale, but they also have an adverse effect on farmers′ livelihoods and the local economy. Improved postharvest management practices, investment in storage infrastructure, and farmer training can all help reduce losses and boost potato farm sustainability, which is crucial for world food security.

## Author Contributions

All authors contributed to the study′s conception, design, material preparation, data collection, and analysis, with Birhanu Ayitegeb Ambaw writing the first draft.

## Funding

No funding was received for this manuscript.

## Disclosure

All authors reviewed and approved the final manuscript.

## Ethics Statement

This study involving human participants was conducted in full compliance with ethical guidelines. The research protocol was reviewed and approved by the Institutional Research Ethics Review Board of Bahir Dar Institute of Technology, Bahir Dar University (Approval No. BiT‐IRERB/036/2024). All participants were adults (≥ 18 years) who provided informed consent prior to participation, and data were anonymized to protect privacy.

## Conflicts of Interest

The authors declare no conflicts of interest.

## Data Availability

The data that support the findings of this study are available from the corresponding author upon reasonable request.
